# T3 enhances neuronal activity in an induced pluripotent stem cell derived model of early human brain development

**DOI:** 10.1530/ETJ-25-0193

**Published:** 2026-01-08

**Authors:** Anna Lopez Marti, Ulgu Arslan, Joris van Dongen, D Sidal Gunduz, Lisa Bauer, Cristina Gontan, W Edward Visser

**Affiliations:** ^1^ Academic Center for Thyroid Disease, Department of Internal Medicine, Erasmus Medical Center, Rotterdam, The Netherlands; ^2^ Department of Viroscience, Erasmus Medical Center, Rotterdam, The Netherlands; ^3^ Department of Developmental Biology, Erasmus Medical Center, Rotterdam, The Netherlands

**Keywords:** thyroid hormone, human-induced pluripotent stem cells, hiPSCs, neuron, MEA, electrophysiology, development, human model

## Abstract

**Background:**

Thyroid hormones are key modulators of brain development, including neuronal activity. Since most studies have been carried out in animal models, information on thyroid hormone signaling in human neurons is scarce. Therefore, human neuronal models that allow electrophysiological assessments are highly warranted.

**Methods:**

We generated human induced pluripotent stem cell (hiPSC)-derived excitatory neurons through Neurogenin-2 (NGN2) overexpression (iNeurons). Astrocytes were added to the cultures to enhance neuron survival. Co-cultures were treated with or without 1 nM triiodothyronine (T3). We assessed thyroid hormone metabolism and transcriptional response in iNeurons with metabolism assays and RT-qPCR of the T3-responsive genes *KLF9* and hairless (*HR*). We investigated the effects of 1 nM T3 on neuronal electrophysiology using multi-electrode arrays (MEAs).

**Results:**

iNeurons presented a dose-dependent induction of *KLF9* (up to nine-fold change) and *HR* (up to eight-fold change). Cultures showed substantial type 3 deiodinase (D3) activity in lysates and intact neurons. MEA recordings showed that T3 treatment increased the overall neuronal activity of the cultures with a ∼2-fold increase in firing rate, ∼3-fold increase in total number of spikes and ∼3.5-fold increase in the number of bursts. Moreover, T3 increased synchronicity by strongly promoting the formation of network bursts (∼14-fold increase), being most prominent between ∼3 and 4 weeks of culture.

**Conclusion:**

Our results indicate that T3 regulates multiple features of neuronal activity in NGN2-differentiated neurons, illustrating its potential to study normal and disordered thyroid hormone signaling in a human model for early brain development.

## Introduction

Thyroid hormones are critical regulators of brain development and function, with triiodothyronine (T3) playing a pivotal role in modulating neuronal differentiation, maturation, and synaptic activity (1, 2, 3). Disrupted thyroid hormone homeostasis has devastating effects on human neurodevelopment, exemplified by the intellectual and motor disability in untreated congenital hypothyroidism and defects in cellular thyroid hormone signaling (2, 4, 5). However, the molecular mechanisms underlying thyroid hormone action in the brain are largely derived from animal models (e.g. rat, mouse, tadpole, and zebrafish) (6, 7, 8, 9); moreover, many animal studies have focused on postnatal brain development with early brain development being largely neglected, while observations in humans clearly indicated the critical dependence on thyroid hormone during early brain development (10). Importantly, animal models do not fully recapitulate the human brain physiology, as cell types are not always conserved and electrophysiology across species is challenging to compare (11, 12).

Recent advances in stem cell biology have enabled the generation of human induced pluripotent stem cell (hiPSC)-derived neurons and brain organoids (13, 14). The utilization of such models has yielded relevant insights for thyroid hormone signaling in physiology and disease (15, 16, 17). However, disadvantages of these models include lengthy culture protocols and being not or less suited for electrophysiological assays. Hence, there is a need for a complementary model that has a rapid culture protocol and is suited for assessing electrophysiology in a reproducible way.

Neurogenin-2 (NGN2)-induced excitatory neurons (iNeurons) offer a rapid and reproducible model of human cortical-like glutamatergic neurons (18). iNeurons have been shown to form functional synapses and exhibit significant electrophysiological activity (19, 20). This model has been optimized to be used in multi-electrode arrays (MEAs), a non-invasive technique that allows us to record spontaneous neuronal activity and observe network connectivity (21, 22).

Here, we report the effects of T3 on various features of neuronal electrophysiology employing MEAs in iNeurons. T3 addition to neuronal cultures caused a maintained increase in neuronal activity in iNeurons. Neuronal activity levels were correlated with intracellular T3 availability regulated by D3. Furthermore, iNeurons contained the key elements of thyroid hormone signaling, demonstrating to be a suitable model to explore T3 action in neural development.

## Methods

### hiPSC culture

hiPSCs containing a doxycycline-inducible NGN2 transgene were grown in mTeSR Plus Medium (STEMCELL Technologies, Canada; #100-0276) on 3 mg/mL matrigel-coated plates (hESC Qualified Matrix, LDVE-free, Corning, USA; #354277) in a humidified incubator at 37°C with 5% CO_2_. The medium was replaced every other day. Once the cells reached at least 80% confluence, they were dissociated using ReLeSR (STEMCELL Technologies, #100-0484) for 5 min at 37°C. The cells were then resuspended in mTeSR Plus, and the required number of cells were transferred to a new matrigel-coated plate.

### Generation of NGN2-hiPSC lines

NGN2-hiPSC lines were generated by targeted integration of a doxycycline-inducible NGN2 cassette into the *AAVS1* safe-harbor locus of the healthy human male WTC-11 hiPSC line (Coriell Repository, GM25256) (23). All experiments were performed using the same NGN2-WTC-11 clone (clone 2) verified by PCR and karyotype analysis (Supplemental Fig. 1 (see the section on Supplementary materials given at the end of the article)). In brief, a donor plasmid (pUCM-AAVS1-TO-hNGN2; Addgene #105840) and a PX458-gRNA-h*AAVS1* vector were co-introduced into hiPSCs via nucleofection. The PX458-gRNA-h*AAVS1* plasmid was generated by cloning the *AAVS1* sgRNA oligonucleotides sg*AAVS1*_sense: 5′-CACCGGGGCCACTAGGGACAGGAT-3′ and sg*AAVS1*_antisense: 5′-AAACATCCTGTCCCTAGTGGCCCC-3′ – into pSpCas9(BB)-2A-GFP (PX458; Addgene, USA; #48138). Nucleofections were performed using the Human Stem Cell Nucleofector Kit 2 (Lonza, Switzerland; VPH-5022), according to the manufacturer’s protocol. 24 h post-nucleofection, cells were selected with 150 ng/mL puromycin for 5 days to enrich for stable integration. Emerging colonies were manually picked and expanded. Genomic DNA was extracted from each clone using the QuickExtract DNA Extraction Kit (Lucigen, USA; QE0905) and screened by PCR genotyping to confirm correct *AAVS1* integration (24). NGN2-hiPSC lines were genotyped using the Infinium Global Screening Array (GSA) (Illumina, USA) to ensure genome stability.

### Differentiation of NGN2 hiPSCs into iNeurons

The day before seeding, plates were coated with 0.01% poly-L-ornithine (PLO) (Merck Life Science, Germany; #P4957-50ML) and incubated at 37°C for at least 3 h. After coating, PLO was removed, and plates were washed three times with sterile water, then air-dried for 30 min. The plates were then coated with 5 μg/mL Biolaminin 521 LN (Biolamina, Sweden; #LN521-05) diluted in Advanced DMEM (Gibco, USA; #12491015) and incubated overnight at 4°C. On the seeding day, hiPSCs were detached with accutase (Millipore, USA; #SCR005) for 5 min at 37°C, followed by collection in PBS, centrifugation at 336 ***g*** for 3 min, and resuspension in mTeSR Plus containing 10 nM Y-27632 dihydrochloride (ROCK inhibitor, Tocris, UK; #1254) and 4 μg/mL doxycycline (Merck Life Sciences, Germany; #D9891-5G). Cell count was performed using the Countess 3 Automated Cell Counter (Invitrogen, USA; #16842556). WTC11-derived astrocytes were added at a 1:1 ratio with hiPSCs on the seeding day.

At 1 day *in vitro* (DIV), the culture medium was completely replaced with advanced DMEM supplemented with 1% N2 supplement (100X) (Gibco, #17502048), 1% MEM non-essential amino acids (Gibco, #11140-050), 1% penicillin/streptomycin, 4 μg/mL doxycycline, 10 ng/mL NT-3 (STEMCELL Technologies, #78074.1), 10 ng/mL BDNF (Prospec, Israel; #CYT-207), and 200 ng/mL mouse laminin (Sigma-Aldrich, USA; #L2020-1MG). At DIV3, the medium was fully replaced with Neurobasal Medium (Gibco, #11570556) supplemented with 2% B27 without vitamin A (50X) (Gibco, #12587010), 1% GlutaMAX-I (100X) (Gibco, #35050-038), 1% penicillin/streptomycin, 1 μg/mL doxycycline, 10 ng/mL NT-3, and 10 ng/mL BDNF (DIV3 medium). B27 supplement was analyzed with LC/MS and ELISA, and no biologically relevant concentrations of T3 were found across at least five batches (data not shown). Two μg/mL cytosine β-D-arabinofuranoside (Ara-C, Merck, #C1768-100MG) was added on DIV3 for 48 h to remove undifferentiated hiPSC and to limit astrocyte overgrowth. When applicable, T3 (Merck Life Science, #T2877-1G) and/or 100 μM iopanoic acid (IOP, Merck Life Science, #14131-100MG) were also added starting at this time point. From DIV3 onward, half of the medium was replaced with fresh DIV3 medium every other day. Doxycycline was added to the medium until DIV14.

### Astrocyte differentiation

Astrocytes derived from WTC-11 hiPSC line were differentiated according to the protocol described by Lendemeijer *et al.* (25). In short, previously differentiated neural progenitor cells (NPCs) were cultured in 20 μg/mL mouse laminin-coated plates (Sigma-Aldrich, #L2020-1MG) in advanced DMEM/F12 supplemented with 1% N2 supplement (100X), 2% B27 without vitamin A (50X), 1% penicillin/streptomycin, 1 μg/mL mouse laminin and 20 ng/mL basic fibroblast growth factor (bFgf) (Sigma-Aldrich, #GF003). To induce astrocyte differentiation, NPCs were cultured with the same basal medium, replacing bFgf with 10 ng/mL bone morphogenic protein 4 (BMP4) (PeproTech, USA; #120-05ET) and 10 ng/mL leukemia inhibitory factor 1 (LIF-1) (PeproTech, #300-05). Medium was changed every other day. After 4 weeks, astrocytes were considered mature and ready to be added to NGN2 neuronal cultures.

### RT-qPCR

iNeurons were collected and RNA was extracted using TRI Reagent (Sigma-Aldrich, #T9424), following the manufacturer’s instructions. Each sample, containing 2,200 ng RNA, was treated with DNAse I (Invitrogen, #18068015) and then reverse transcribed using the Transcriptor High Fidelity cDNA Synthesis Kit (Roche, Switzerland; #5081955001), as per the manufacturer’s guidelines. The qPCR reaction was performed using the FastStart Universal Probe Master (ROX) (Roche, #4914058001) on a QuantStudio 7 Flex Real-Time PCR System (Applied Biosystems, USA; Thermo Fisher, USA). TaqMan gene expression assays included *KLF9* (Thermo Fisher, Assay ID Hs00230918_m1), hairless* (HR)* (Thermo Fisher, Assay ID Hs00218222_m1), *THRA* (Thermo Fisher, Assay ID Hs00268470_m1), *SLC16A2* (MCT8) (5′-CCA​TAA​CTC​TGT​CGG​GAT​CCT​C-3′ and 5′-ACT​CAC​AAT​GGG​AGA​ACA​GAA​GAA​G-3′ with probe 5′FAM-ATACCCATCGCGAGGGCTCCGA-TAMRA-3′) and *GAPDH* (Thermo Fisher, Assay ID Hs04420632_g1) and *ACTB* (β-actin) (Thermo Fisher, Assay ID Hs03023943_g1) as the housekeeping genes. For *VGLUT2*, 5′-TCA​GCA​GCC​AGA​GTG​CAT​TA-3′ and 5′-AGG​TCA​CAC​CCT​CAA​CAA​GTC-3′ were used, and for GAD1, primers 5′-GCT​CAA​GAT​CTG​CGG​CTT​CTT-3′ and 5′-GGA​AAG​CAG​GTT​CTT​GGA​GGA-3′. *VGLUT2* and *GAD1* were amplified with GoTaq qPCR master mix (Promega, USA; #A6001). Foldchanges were calculated using the ΔΔCT method. Data analysis was conducted using the QuantStudio Software v1.7.2 and GraphPad Prism (GraphPad Software, Inc., USA).

### Immunocytochemistry

Neuronal cultures were grown on coverslips and fixed using 4% paraformaldehyde in PBS. The coverslips were then rinsed three times for 5 minutes with PBS and blocked for 1 hour in a staining buffer containing 0.05 M Tris, 0.9% NaCl, 0.25% gelatin, and 0.5% Triton-X-100 (pH 7.4). Primary antibodies were incubated overnight at 4°C in the same staining buffer. The following primary antibodies were used: MAP2 (1:200, guinea pig polyclonal, Synaptic Systems, #188-004), NeuN (1:100, rabbit monoclonal, Sigma-Aldrich, #ZRB377-25UL), CD44 (1:200, rat monoclonal, Sigma-Aldrich, #SAB4700188-100UG), CTIP2 (1:100, rabbit polyclonal, Abcam, UK; #ab28448), Syn1 (1:100, rabbit polyclonal, Synaptic Systems, #106-103), PSD95 (1:100, mouse monoclonal, Invitrogen, #MA1-046) and S100β (1:100, mouse monoclonal, Sigma Aldrich, #S2532-.2ML). To remove unbound antibodies, coverslips were washed three times for 5 minutes each with PBS between antibody incubations. The secondary antibodies used were A647 donkey anti-guinea pig (1:200, Bio-connect, #AB_2340476), A488 goat anti-rabbit (1:100, Abcam, #ab150077), Cy3 donkey anti-rat (1:200, Bio-connect, Netherlands; #AB_2340666), and A555 goat anti-mouse (1:100, Life Technologies, #A21422). All secondary antibodies were incubated for 1 hour at RT. Samples were washed three more times in PBS to remove excess antibody. Samples were embedded in Mowiol 4–88 (Carl Roth, Germany; #0713) before being imaged using a Zeiss LSM700 (Zeiss, Germany) or Stellaris 5 (Leica, Germany) confocal microscopes. Image processing was conducted with ImageJ.

### Radioactive assays

#### Whole cells

NGN2 neuronal co-cultures were grown for 24 days. On DIV24, 1 nM (2 × 10^6^ cpm) [^125^I]-T3 was added to the culture. In the samples treated with IOP, 100 μM IOP was added. Cells were incubated with radioactive iodine for 24 h at 37°C and 5% CO_2_. Then, 100 μL medium were collected and mixed 1:1 with ice-cold 0.1% acetic acid in acetonitrile. Protein precipitation of the samples was carried out on ice for 60 min followed by centrifugation at 37,318 ***g*** at 4°C for 15 min. 100 μL supernatant were mixed with 125 μL 20 mmol/L ammonium acetate and analyzed by UPLC (waters). Radioactivity was measured by a RAMONA HPLC-LS pump scintillation detector (Elysia-Raytest, Germany).

#### Lysates

Deiodinase type 3 activity was measured as previously published (26, 27). In summary, NGN2 co-cultures were lysed in PED10 buffer (50 mmol/L sodium phosphate (pH 7.2) with 1 mmol/L EDTA and 20 mmol/L DTT). Protein concentration was measured with Bio-Rad Protein Assay Kit II (Bio-Rad, USA; #5000002). 50 μL the cell lysates were incubated in 50 μL PED10 buffer containing 1 nmol/L (2 × 10^5^ cpm) [^125^I]-T3 and 1 nmol/L unlabeled T3 at 37°C for 60 min. In samples treated with IOP, 20 μM was added to the mix. The reaction was stopped by adding 125 μL ice-cold 0.1% acetic acid in acetonitrile and mixed with vortex. Samples were kept on ice for 60 min to allow protein precipitation and then centrifuged for 15 min at 37,318 ***g*** and 4°C. 125 μL supernatant were collected and mixed with 100 μL 20 mmol/L ammonium acetate and analyzed by UPLC paired to the RAMONA HPLC-LS pump scintillation detector. The activity was corrected for protein content and incubation time.

### Multi-electrode arrays (MEAs)

NGN2 hiPSCs were plated and differentiated to iNeurons following the established protocol in a CytoView MEA plate (Axion BioSystems, USA; #M384-tMEA-24W). On DIV14, the culture medium was replaced with BrainPhys Neuronal Culture Medium (STEMCELL Technologies, #05790), supplemented with 2% B27 supplement minus vitamin A (50X), 1% GlutaMAX-I (100X), 1% penicillin/streptomycin, 10 ng/mL NT-3 and 10 ng/mL BDNF. Starting from DIV15, spontaneous neuronal activity was recorded for 5 minutes at 37°C and 5% CO_2_ using the Maestro Edge system (Axion BioSystems). Data analysis was performed using AxIS Navigator 3.9.1, Neural Metric Tool software (Axion BioSystems), and GraphPad Prism (GraphPad Software, Inc., USA). Spike detection was carried out using adaptive threshold crossing at 6 × SD, while the inter-spike interval (ISI) threshold was applied to identify bursts (minimum number of spikes = 5; maximum ISI = 100 ms) and network bursts (minimum number of spikes = 50, with at least 35% of electrodes participating).

### Statistical analysis

All statistical analyses were conducted using the GraphPad Prism (version 9). In RT-qPCR and metabolism experiments, analyses were performed using duplicates or triplicates from 3 to 4 independent neuronal differentiations. For MEA analysis, each data point represents neuronal activity from a single well (*n*) from three independent batches. When two conditions were analyzed, unpaired *t*-test was used. When three or more conditions were tested, one-way ANOVA, two-way ANOVA or Kruskal–Wallis tests were used. For all experiments, normality was validated with Shapiro–Wilk test. For all testing, the level of significance was set at a two-tailed *P* < 0.05, and the error bars represent the standard error of mean (SEM).

## Results

### iNeurons as a model to investigate thyroid hormone signaling in human brain development

**Figure 1:**
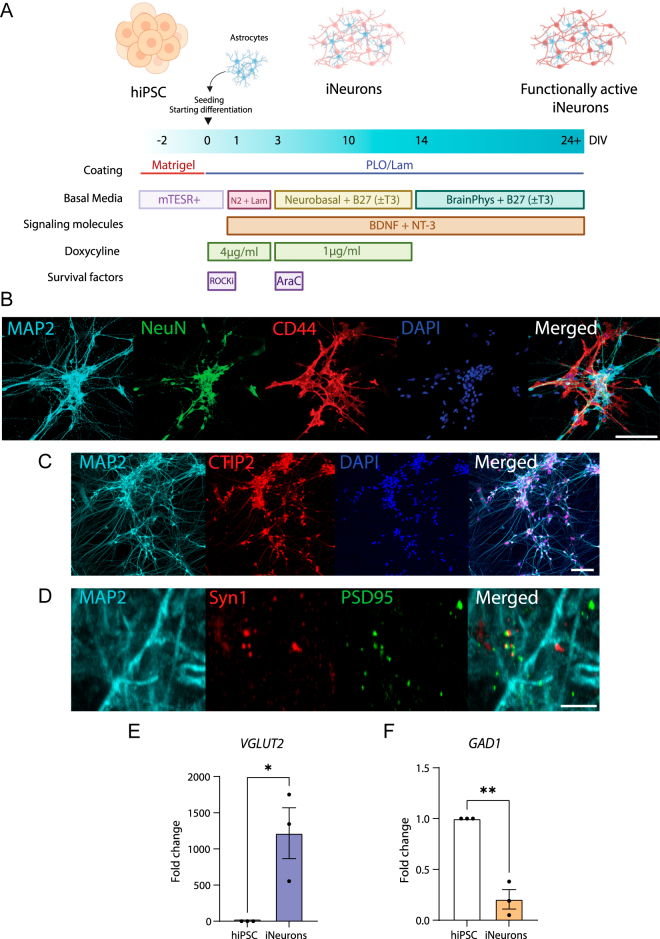
iNeurons as a model for thyroid hormone action in human brain development. (A) Schematic representation of the differentiation protocol from NGN2 hiPSC to iNeurons. Coating, basal media and various factors are depicted. hiPSC were cultured with doxycycline to induce expression of the NGN2 transgene. At DIV0, hiPSC-derived astrocytes are added to support the neuronal culture. Cells were cultured with or without T3 from DIV3. At DIV10, neuronal cultures are generated. At DIV14, the medium is changed from Neurobasal to BrainPhys to achieve functionally active cultures around DIV24. (B, C, D) Representative images of NGN2 co-cultures at DIV10-15. (B and C) Cultures present mature neuronal markers MAP2 (light blue) and NeuN (green) and the deep-layer cortical marker CTIP2 (red). Astrocytes are integrated in the culture as indicated by the CD44 (red) positive cells. Scale bar = 100 μm. (D) Co-localization of Synapsin 1 (red) and PSD95 (greed) indicate excitatory synapses. Scale bar = 5 μm. Gene expression analysis by qPCR of the (E) glutamate transporter *VGLUT2* and the (F) GABAergic marker *GAD1*. *n* = 3 independent experiments, each including three technical replicated, unpaired *t*-test. Error bars = SEM, *P < 0.05, **P < 0.01, ***P < 0.001, ****P < 0.0001.

We employed inducible Neurogenin-2 (NGN2) overexpression (28) (iNeurons), which rapidly and reproducibly differentiates hiPSC into neurons in 10 days (Fig. 1A). To ensure optimal survival and function of the neuronal cultures, hiPSC-derived astrocytes were added to the cultures according to the established protocol (Fig. 1A and B). By 10 days, iNeurons expressed markers of maturing postmitotic neurons, such as MAP2 and NeuN (29, 30). CD44-positive astrocytes were integrated in the culture. There were no cells co-expressing neuronal and astrocytic markers (Fig. 1B). Our protocol generated a homogenous population of CTIP2-positive deep-layer cortical neurons (31) (Fig. 1C) that could form Synapsin1-PSD95 excitatory synapses (32) (Fig. 1D). Furthermore, our cultures strongly expressed the vesicular glutamate transporter *VGLUT2* (Fig. 1E). The GABAergic marker *GAD1* was virtually absent and had even lower expression compared to the hiPSC stage (Fig. 1F). Together, these results confirm the excitatory nature of our iNeurons in agreement with previous studies (33, 34).

### iNeurons present key elements of thyroid hormone signaling

**Figure 2:**
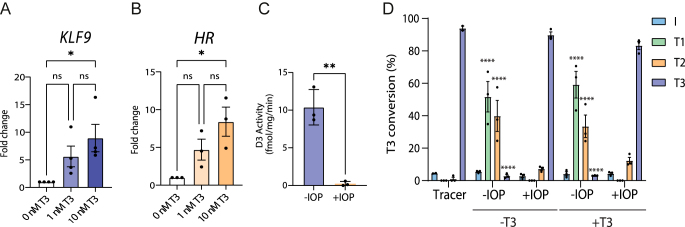
iNeurons respond to and metabolize T3. Gene expression analysis by qPCR of T3-responsive genes (A) *KLF9* and (B) *HR*. *n* ≥ 3; at least three independent experiments comprising three technical replicates. *KLF9* = Kruskal–Wallis, *HR* = one-way ANOVA, Tukey’s multiple comparisons. (C) D3 activity from iNeuron lysates treated with or without iopanoic acid (IOP). *n* = 3; three independent experiments with two technical replicates, unpaired *t*-test. Error bars = SEM. (D) Conversion of T3 to other metabolites in whole cells. Cells were exposed to radioactive T3 (tracer) for 24 h. T3 was converted to T2 and T1. Cells were treated with or without T3 and with or without IOP. *n* = 3; three independent experiments with technical duplicates, two-way ANOVA with Dunnett’s multiple comparisons (tracer as control). Error bars = SEM, *P < 0.05, **P < 0.01, ***P < 0.001, ****P < 0.0001.

Thyroid hormones exert their action by entering the nucleus and binding to the thyroid hormone receptors (TRs). To confirm that our iNeuron cultures respond to thyroid hormone, we cultured them for 10 days in the presence of 0, 1 and 10 nM T3. We performed RT-qPCR for *KLF9* and *HR*, two widely used T3-responsive genes in multiple tissues and species (1, 35). A non-significant but consistent trend toward increased *KLF9* and *HR* expression with higher T3 concentration was observed, with the 1 nM T3 concentration already inducing a response (Fig. 2A and B). In line with early studies in *ex vivo* rat brain slices that showed optimal T3 effects in the low nanomolar range, we selected 1 nM T3 to investigate its effects in our neuronal cultures (36, 37).

Another essential signaling element is the metabolism of thyroid hormone, with deiodinases removing iodine atoms from thyroid hormones. To assess whether our model mimics the early stages of the developing human brain, which has considerable type 3 deiodinase (D3) activity (38, 39), we investigated if our model presented D3 activity. First, we measured D3 activity in lysates from iNeurons. We observed substantial D3 activity in the lysates, which was almost completely reduced when lysates were treated with IOP, a known compound used to block deiodinase activity (40) (Fig. 2C). Next, we validated and extended this finding by measuring deiodination in intact cells, requiring both transmembrane transport and metabolism of T3. Assays using radiolabelled T3 ([^125^I]-T3) revealed that iNeurons are able to transport and convert T3 into T2, and also T2 into T1, after 24 h of exposure to [^125^I]-T3 (Fig. 2D). T3 metabolism was similar between cells cultured with or without T3. This conversion was reduced by ∼90% upon addition of 100 μM IOP, which confirms its deiodinating nature. All in all, our findings indicate that iNeurons express functional transporters, D3 and TRs, as essential elements of thyroid hormone signaling.

### Thyroid hormone increases electrophysiological activity in iNeurons

**Figure 3:**
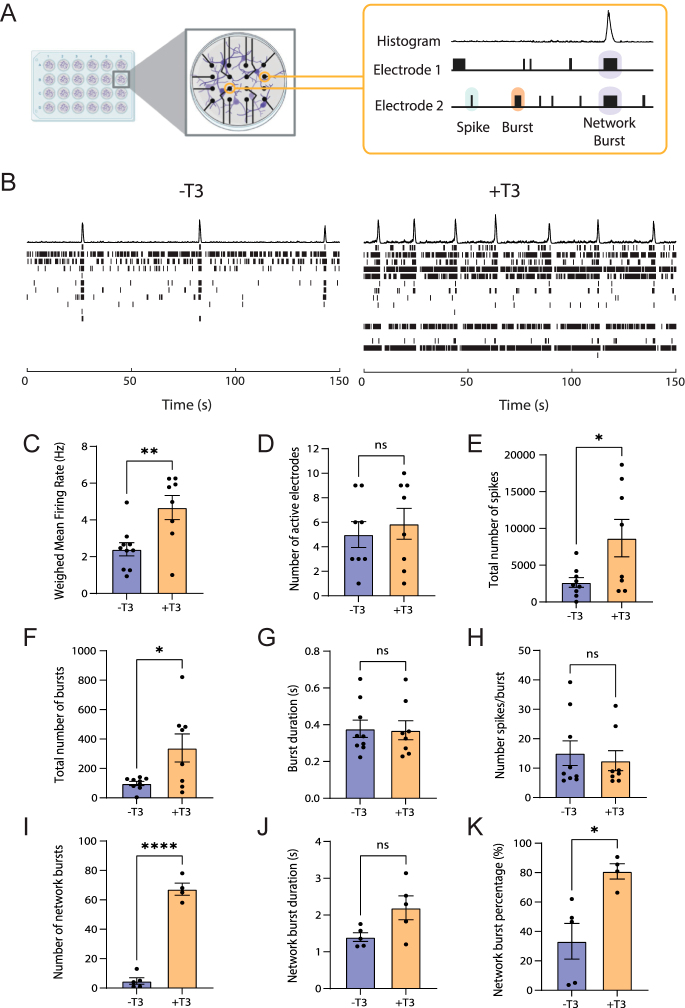
T3 increases neuronal activity. (A) Schematic representation of MEAs. Electrodes are embedded in the cell culture plate and record spontaneous neuronal activity; single spikes (blue), trains of spikes or bursts (orange) and network bursts (purple). The histograms indicate network bursts. (B) Representative raster plots showing 150 s of activity of NGN2 cultures at DIV26. Each row represents an electrode. (C) Quantification of weighed mean firing rate, *n* = 8–10 (individual wells), unpaired *t*-test; (D) number of active electrodes, *n* = 8–10, unpaired *t*-test; (E) total number of spikes, *n* = 9, unpaired *t*-test; (F) total number of bursts, *n* = 9, unpaired *t*-test; (G) burst duration, *n* = 9, unpaired *t*-test; (H) number of spikes/burst, *n* = 9, Mann–Whitney test; (I) total number of network bursts, *n* = 5, unpaired *t*-test; (J) network bursts duration, *n* = 5, unpaired *t*-test; and (K) network bursts percentage, *n* = 5, unpaired *t*-test. Error bars = SEM, *P < 0.05, **P < 0.01, ***P < 0.001, ****P < 0.0001.

During brain development, thyroid hormone regulates multiple processes, including neuronal activity, cognition and behavioral changes (41, 42, 43). Therefore, we investigated the effects of T3 on the electrophysiological properties of our cultures using MEA. MEAs are a non-invasive technique that allows to record three different types of neuronal activity: a) spikes, random neuronal activity (Fig. 3A, blue box); b) bursts, localized trains of spikes (Fig. 3A, orange box); and c) network bursts, bursts detected simultaneously in at least 35% of the electrodes (Fig. 3A, purple box). At DIV26, in T3-treated iNeuron cultures, we observed an overall increase in the activity of the cultures (Fig. 3B). Addition of T3 to the cultures resulted in a ∼two-fold increase in the weighed mean firing rate and ∼three-fold increase in the number of total spikes, while the number of active electrodes remained similar (Fig. 3C, D, E). The total number of bursts was ∼3.5-fold higher in cultures exposed to T3, indicating enhanced neuronal activity when T3 is present (Fig. 3F). The burst duration and the number of spikes per burst were comparable in the two conditions (Fig. 3G and H). Network bursts were the parameter mostly affected by T3 treatment with the number of network bursts increasing ∼14-fold in T3-treated cultures and remaining active ∼1.5-fold longer (Fig. 3I and J). In addition, when T3 was present in the culture, the majority of the spikes detected during the recordings contributed to network bursts as represented by the network bursts percentage (Fig. 3K).

### Neuronal activity levels inversely correlate with D3 activity

**Figure 4:**
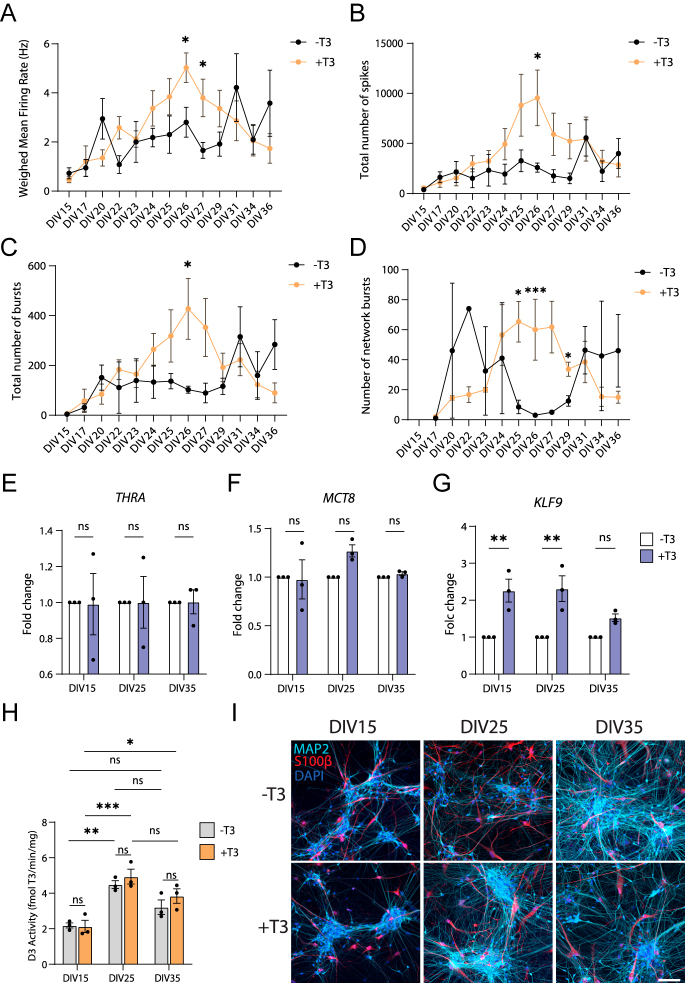
T3 maintains an increased neuronal activity over time. Cultures were treated with or without 1 nM T3 for up to 36 days (DIV) and neuronal activity was recorded several times. T3-treated cells presented an overall increase in the mean firing rate (A), total number of spikes (B), total number of bursts (C) and number of network bursts (D). *n* = 4–10, unpaired *t*-test. (E, F, G) Temporal gene expression analysis by qPCR of *THRA* (E), *MCT8* (F) and *KLF9* (G). (H) D3 activity of co-cultures over time. (I) Representative images of the ratio of iNeurons (MAP2, light blue) to astrocytes (S100β, red) in different time points. Error bars = SEM, *P < 0.05, **P < 0.01, ***P < 0.001, ****P < 0.0001.

To assess the dynamics of T3 effects on electrophysiology during longer time periods, we recorded spontaneous neuronal activity from DIV15 until DIV36. This showed a gradual increase of electrophysiological activity over time, peaking between DIV24 and DIV29 (Fig. 4A, B, C, D). Neurons exposed to T3 showed an increase in the mean firing rate (Fig. 4A), the total number of spikes (Fig. 4B), total number of bursts (Fig. 4C) and the number of network bursts (Fig. 4D), indicating a progressive and robust maturation of the neural networks. In contrast, the cells without T3 showed a more inconsistent behavior.

Next, we explored if the T3-induced increase in neuronal activity was caused by changes in the responsivity to T3. We analyzed different elements of thyroid hormone signaling in our co-cultures at three different time points: DIV15, when neuronal activity is low; DIV25, when cultures near the peak of activity; and DIV35, when activity decreases again. There were no differences in the expression of thyroid hormone receptor alpha (*THRA*) (Fig. 4E) and thyroid hormone transporter *MCT8* (*SLC16A2*) (Fig. 4F), suggesting a stable expression of the main receptor and transporter irrespective of time and T3 concentration. However, *KLF9* induction decreased at DIV35, where *KLF9* expression was comparable between cells treated with and without T3 (Fig. 4G). D3 activity significantly increased in DIV25 compared to DIV15 to then decrease at DIV35 to levels between DIV15 and DIV25 (Fig. 4H). These changes in D3 activity suggest differences in the intracellular availability of T3, which could explain the differences in *KLF9* induction over time.

Finally, we questioned if the ratio of iNeurons to astrocytes in our co-cultures changed over time or by T3, which could also explain the differences in electrophysiological activity. No apparent changes were observed in the amount of astrocytes (positive for S100β) between cells treated with or without T3, and also not between the different time points (Fig. 4I).

All in all, our results suggest that T3 is involved in the maturation of human neurons by enhancing neuronal activity and promoting synchronicity with D3 activity fine-tuning intracellular bioavailability of T3.

## Discussion

In this study, we report a model harboring essential elements of thyroid hormone signaling, which can be used to investigate the effects of T3 on the electrophysiology of hiPSC-derived neurons.

A principal finding was that MEA recordings showed that 1 nM T3 exposure in the cultures strongly enhanced many features of neuronal activity, particularly stimulating consistent network bursting. These observations are in line with a previous study that employed hiPSC-derived neural cells with a much higher T3 concentration (100 nM), which showed an increased number of spikes and bursts upon T3 addition (15). Bursting and synchronicity are intrinsic properties of early brain development both in rodents and in humans, as they promote neuronal communication (44, 45). Our data suggests that T3 is involved in the regulation of neural activity and synchronicity in early stages of human neurodevelopment with a key role for D3 activity to regulate intracellular bioavailability of T3.

T3-induced increase in activity is likely caused by an increase in responsiveness to T3. As D3 activity changes over time, while expression of thyroid hormone receptor and transporter remained stable, intracellular concentrations of T3 are mostly regulated by D3. We observed changes in D3 activity, reaching a maximum at DIV25 and decreasing again at DIV35. Thus, the reported D3 fluctuations presumably influence intracellular T3 availability and may explain the increase in activity between DIV24-DIV27. Both T3-dependent gene expression and electrophysiological activity showed a delayed response compared to the D3 activity fluctuations, as we observed the potential effects of high D3 activity (thus low T3 levels) at later time points (DIV29 onward). Hence, T3 might influence electrophysiological activity by triggering gene expression of downstream of T3-responsive genes involved in neural activity, although the direct correlation between T3-dependent genes and neural activity remains unclear. Synaptogenesis has been reportedly found to be increased in the presence of T3 in rat neurons *in vitro*, which correlated with increased calcium waves, indicating higher synaptic vesicle release (46). Other studies in rats showed that T3 treatment resulted in increased excitability of neurons due to increased sodium currents (47). Thus, our model may represent a useful tool to study electrophysiological properties of low, potentially more physiologically relevant, T3 concentrations. Future studies (e.g., measuring T3-dependent gene expression of synaptic genes by RNA-seq or quantifying synapses) are needed to reveal the molecular mechanisms underlying the observed electrophysiological phenomena.

With the complexity of the human brain, different human models may capture different aspects. Previously, neural networks and 3D brain organoids have provided relevant insights in the expression of key players of thyroid hormone signaling and in the functional role of thyroid hormone transporter MCT8 (15, 16, 17). However, neuronal function as assessed by electrophysiology is intrinsically difficult to quantify in such 3D brain organoids. Hence, our NGN2-differentiated neuronal model could complement such organoid models as, thanks to its 2D-nature, our model facilitates the study of fundamental molecular mechanisms and is also easy to culture on devices, such as MEA, thereby facilitating the study of electrophysiology, unlike other more complex models.

Our study also presents limitations. First, our model requires astrocytes that support the neuronal networks. Although this positively affects their electrophysiology, and also better mimics the human brain, astrocytes are also exposed to T3. Therefore, we cannot rule out that the effects on electrophysiology are (partially) mediated through T3 that alters properties of astrocytes (25, 48). Second, although human iPSCs are an excellent tool that allows us to better understand human early brain development, it remains important to realize that *in vitro* models fall short in representing the complex brain physiology (49). For example, NGN2-based differentiation protocols might skip developmental steps that would affect the nature of the neurons produced. In addition, the current protocol generates excitatory neurons, whereas the brain has many other cell types, including inhibitory neurons and myelinating oligodendrocytes *in vivo* (50, 51). Finally, our protocol does not maintain a self-organizing 3D structure and lacks a vascular system and other relevant cell types present in the brain, which are key features of brain physiology *in vivo*.

In summary, we present a hiPSC-based neuronal model, which contains key elements of thyroid hormone signaling and allows to assess many features of neuronal electrophysiology. This platform can be used to model normal and disordered thyroid hormone signaling in early human brain development.

## Supplementary materials



## Declaration of interest

The authors declare that there is no conflict of interest that could be perceived as prejudicing the impartiality of the work reported. WEV is on the Editorail board of the *European Thyroid Journal*. He was not involved in the peer review process of this paper on which he is listed as an author.

## Funding

WEV acknowledges funding from Erasmus MC (fellowship award) and from Netherlands Organisation for Health Research and Development (ZonMw) (VIDI).

## Author contribution statement

ALM and WEV designed the study and wrote the manuscript. ALM, UA, DSG, LB, CG and WEV conceived the methodology. ALM, UA, JvD and DSG performed and analyzed the experiments. CG generated the NGN2 hiPSCs. All authors revised and approved the manuscript.

## Data availability

All data are available in the main text. The raw data used in the analysis are available upon request.
