# Supplementary material for: T3 enhances neuronal activity in an induced pluripotent stem cell derived model of early human brain development
Source: Eur Thyroid J. 2026 Jan 8;15(1):ETJ250193. doi: 10.1530/ETJ-25-0193 (PMC13138590; doi:10.1530/ETJ-25-0193)
Supplement: Supplementary file 1 [file supplementary_figure_1.pdf]

A.

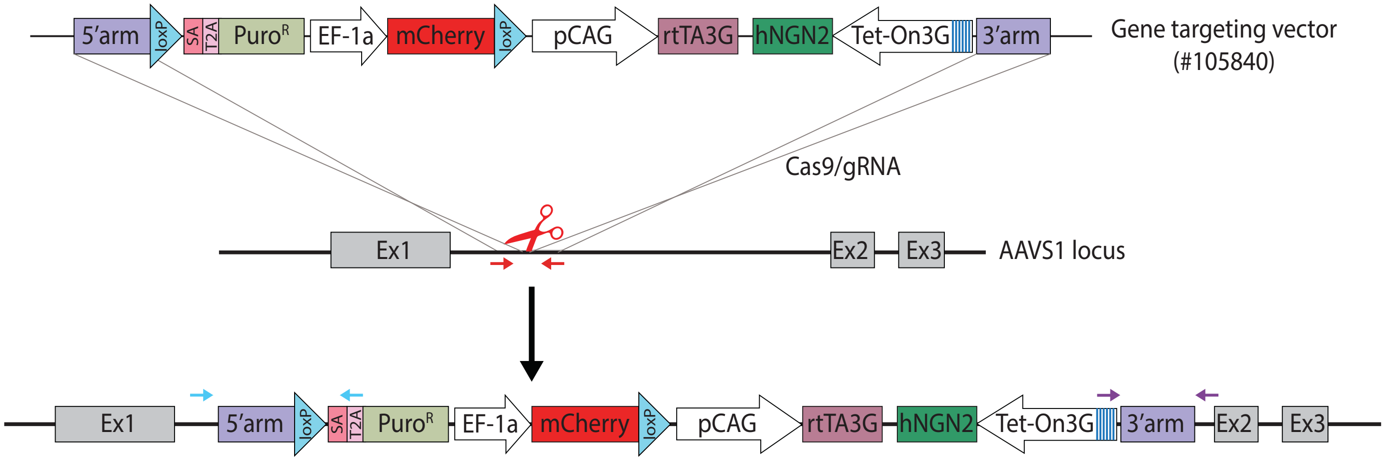

B.

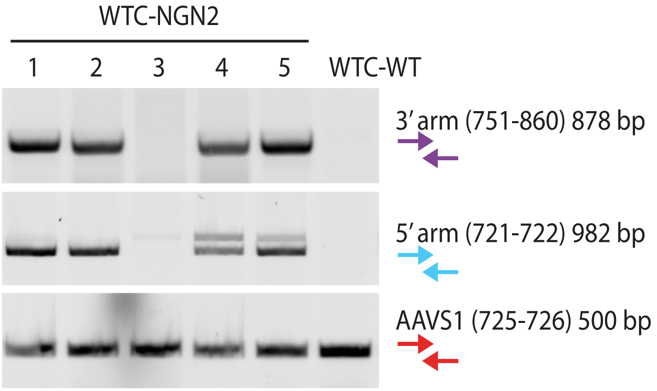

C.

Primer	Sequence (5' - 3')
5' arm FW	CACTTTGAGCTCTACTGGCTTC
5' arm RV	CATGTTAGAAGACTTCCTCTGC
3' arm FW	GGGCTCAGTCTGAAGAGCAG
3' arm RV	TGTGGGGTGGAGATATCAGC
AAVS1 FW	TTCGGGTCACCTCTCACTCC
AAVS1 RV	GGCTCCATCGTAAGCAAACC
